# A Novel Epilepsy Phenotype in a Young Girl With a Pathogenic *SETD5* Gene Variant

**DOI:** 10.1177/08830738251345038

**Published:** 2025-06-04

**Authors:** Davide Alessi, Mariapaola Schifino, Giovanna Traficante, Giulia Gori, Emanuele Bartolini

**Affiliations:** 1 Department of Developmental Neuroscience, IRCCS Stella Maris Foundation, Pisa, Italy; 2 Department of Clinical and Experimental Medicine, University of Pisa, Pisa, Italy; 3 Medical Genetics Unit, Meyer Children's Hospital IRCCS, Florence, Italy

**Keywords:** electroencephalogram, epilepsy, genetics, neurodevelopmental disorders

## Abstract

Recent studies suggest a possible association between variants in *SETD5* and epilepsy, particularly in individuals with intellectual disability and developmental delay. However, the current understanding of *SETD5* function in epilepsy is limited. We describe a 6-year-old girl harboring a pathogenic *SETD5* gene variant, disclosed in early infancy by whole exome sequencing that was performed for global developmental delay. Her neurologic phenotype evolved during follow-up to include focal and generalized seizures as well as an overt neurodevelopmental disorder, characterized by receptive-expressive language difficulties with speech disorder and mild cognitive impairment. Her clinical picture was also characterized by recurrent urinary tract infections in a duplex collecting system due to a concomitant and unrelated *GREB1L* gene variant. Our findings confirm that epilepsy may arise after *SETD5* variants, with subtle clinical manifestations that may overlap with behavioral phenomena in children who also exhibit cognitive and behavioral comorbidities.

Epilepsy is a complex neurologic disorder that has been linked to many genetic variants, but the specific role of some genes, including *SETD5*, remains under investigation. The current understanding of *SETD5*’s role in seizure development and of the associated epilepsy phenotype is limited.^1,2^ To date, most data on SETD5 derive from patients investigated for neurodevelopmental disorders, and only a few reports of seizures are available.

Herein we describe a 6-year-old girl harboring a pathogenic *SETD5* variant, disclosed in early infancy by whole exome sequencing.

## Case Report

The proband was born at term, after an uneventful pregnancy. At 2 months of age, she was investigated for urinary infection, disclosing a duplex collecting system and caliectasis. The same abnormalities were reported in her maternal grandmother. Mild dysmorphic features were also noted (arched eyebrows, epicanthus, palpebral ptosis, broadened nasal bridge, thin lips, mild retrognathia).

Global neurodevelopmental delays were noted in early infancy, especially in language expression and comprehension. Similarly, her mother was also affected by language delay and learning difficulties during childhood. Unassisted walking was acquired at 18 months of age.

She was investigated early by screening for neurometabolic disorders and congenital infections as well as by array-CGH, which were unrevealing. Whole exome sequencing (WES) unveiled 2 heterozygous variants in *SETD5* and *GREB1L*, both maternally inherited. The *SETD5* variant was classified as pathogenetic, the *GREB1L* variant as VUS (variant of uncertain significance) according to the American College of Medical Genetics and Genomics (ACMG) guidelines.^3^

The *GREB1L* variant [NM_001142966:c.3196C > T; p. (Arg1066Cys)] has already been reported elsewhere as linked to renal abnormalities.^4^

The *SETD5* novel variant [NM_001080517:c.2644C > T; p. (Arg.882*)] introduces a premature stop codon, suggesting a loss of function and haploinsufficiency as the common disease-causing mechanisms.

The patient's mother inherited the *GREB1L* variant from her mother, whereas the *SETD5* variant occurred de novo. At 3 years of age, the proband developed waxing behavioral issues with temper tantrums, motor hyperactivity, and attention liability. Borderline cognitive functioning was detected by the Wechsler Preschool and Primary Scale of Intelligence IV (WPSSI-IV) intelligence scale. The Child Behavior Checklist demonstrated scores in the clinical range for affective disorders, anxiety disorders, somatic problems, attention-deficit hyperactivity disorder (ADHD), oppositional defiant disorder, and conduct problems. A 3-tesla brain magnetic resonance imaging (MRI) showed mild enlargement of the cisterna magna.

**Figure 1.**
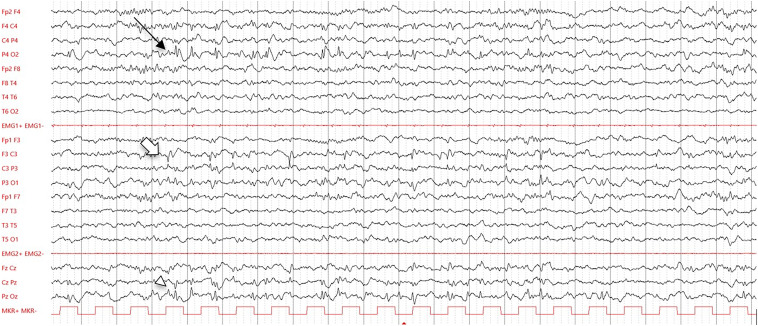
Electroencephalography (EEG) interictal features (10-20 International System, 20 s/page, low-pass band filter: 70 Hz, high-pass-band filter: 1 Hz, gain: 200 mcV/cm). Spike-and-wave discharges, synchronous over the right (thin arrow) and midline (arrowhead) parieto-occipital regions and asynchronous over the left centroparietal areas (arrow).

Her mother described also episodes of blank staring, followed by spreading hypertonia with upward ocular deviation, recurring on a monthly basis, first interpreted as oppositional behaviors in a child with frail emotional regulation. However, episodes increased in frequency, associated with motor phenomena, that is, left head turning and axo-rhizomelic tonic posturing leading to falls. At 4 years of age, we performed prolonged video electroencephalographic (EEG) monitoring, which revealed abundant posterior spike-and-wave discharges, mainly centered in the right parieto-occipital areas, and independent left centroparietal spikes (Figure 1). We interpreted her paroxysmal disturbances as focal impaired-consciousness seizures and introduced levetiracetam 60 mg/kg/d. However, as behavioral worsening was reported, we switched treatment to valproic acid 30 mg/kg/d. After 14 months, the patient is seizure-free and the EEG normalized. Sporadic temper tantrums are still present, yet her behavioral profile has remarkably improved.

## Discussion

*SETD5* gene encodes for a methyltransferase widely expressed in the brain, proven to control histone acetylation, regulate transcription, and contribute to cell cycle progression and embryonic development. Frameshift/nonsense SETD5 variants are extremely rare in controls and intolerant to loss of function mutations (low Residual Variation Intolerance score), strongly supporting pathogenicity of the variant we identified.^1^ This aligns with established SETD5 loss-of-function mechanisms causing neurodevelopmental disorders.

In fact, haploinsufficiency of *SETD5* is associated with intellectual disability, language delay, dysmorphic features, as well as facial, cardiac, genitourinary, gastrointestinal, skeletal abnormalities, and short stature. The gene is also included in the 3p25.3 deletion syndrome, indicating its significant pathogenic role in this condition.^2^ In a systematic review by Fernandes et al,^5^ 23.8% of cases with *SETD5* variants displayed autistic features, and 92.8% was associated with intellectual disability.

An increasing amount of evidence suggests an association between *SETD5* variants and epilepsy, with incomplete penetrance. Fernandes et al^5^ reported seizures in 23.8% of *SETD5* patients (n = 10/42), without specific characterization. Anecdotal reports suggest a variable spectrum of epilepsy presentation. Apparently, some cases may present with early-onset epilepsy.^6,7^ In particular, Albuz et al^6^ reported drug-resistant convulsive seizures since 2 months of age in a child with a copy number variation (deletion in 3p25.3) including *SETD5*. Similarly, Kuechler et al^1^ reported seizures in patients with copy number variations spanning over the *SETD5* gene, describing fever-induced seizures. Kobayashi et al^7^ described a male patient (variant c.2347-7A > G) affected by West syndrome, who presented with infantile spasms starting at 7 months of age but with seizure freedom following treatment with lamotrigine. This variant affected the intron 16 of *SETD5* gene, and resulted in a premature stop codon in the mutant transcript. Halvardson et al^8^ reported a stop-gain *SETD5* variant in a patient with intellectual disability and myoclonic seizures, although the phenotype could be influenced by a co-occurring mutation in *ERC2*.

Instead, Manokaran et al^9^ describe a girl [c.3001C > T; p. (Arg.1001*)] who at age 9 years developed focal epilepsy and posterior EEG abnormalities, partly resembling the features of our patient. Reported seizures usually started with brief headache and dizziness followed by a scared look and screaming, eye deviation to the left, vomiting and coughing, and complex hallucinations. Interictal EEGs showed abundant sleep-activated right occipitotemporal spike/sharp waves. After 4 years, abnormalities became more right hemispheric and then generalized, possibly representing secondary bilateral synchrony. She also exhibited mild cognitive decline over time. Epilepsy was drug-resistant; eventually, she was successfully treated with a ketogenic diet.

Our patient presented with focal-impaired consciousness seizures that were initially difficult to diagnose, considering the concomitant behavior disorder that may yield nonepileptic spells. Nevertheless, motor phenomena with left head turning and axial posturing leading to falls were hardly explainable, and the electroencephalographic assessment allowed to identify abundant clear-cut epileptiform discharges. We could not capture an event by video-EEG monitoring at first assessment, and the introduction of valproate promptly led to seizure cessation.

The patient also exhibited a variant of uncertain significance in *GREB1L*, which could be interpreted as “Hot” for the location in a critical protein region, the absence in control databases (gnomAD), and the co-occurrence with phenotypic features recurring in carriers of the same family.^10^ Hence, it is likely responsible for the duplex collecting system and caliectasis. Mutations of *GREB1L* are frequently associated with renal abnormalities. Schrauwen et al^11^ conducted a comprehensive review of documented *GREB1L* variants, identifying a total of 49 variants. In the review, the investigated population exhibited a range of multisystemic malformations affecting the kidneys, bladder, uterus, and ears, as well as primarily skeletal abnormalities. Notably, there was phenotypic variability within the affected families, and reduced penetrance was observed in 50% of cases. Apparently, no correlation between *GREB1L* variants and epilepsy has been described in the literature.

Summarizing, data suggest that variants in *SETD5* are associated with a wide clinical spectrum and a reduced penetrance and expressivity especially in females. Healthy carrier mothers of affected children have been described.^12^ Our patient's mother, carrying the same mutation, shared some clinical features with the daughter, such as developmental delay and learning difficulties, but did not develop epilepsy or significant cognitive impairment. Therefore, it is crucial to collect and share data to better understand the role of *SETD5* in neurodevelopment and neurophysiology. Although a single report cannot provide deeper insights into the SETD5-related epilepsy, our case report describes a partially novel phenotype associated with *SETD5*, expanding the known clinical spectrum by the description of insidious epileptic seizures that need to be distinguished by behavioral phenomena. Of note, the diagnosis of epilepsy allowed us to treat appropriately, stopping seizures. This finding underscores the importance of detailed phenotypic characterization to improve diagnostic accuracy and advance our understanding of genotype-phenotype correlations. Further studies, including functional analyses and additional case reports, are needed to elucidate the underlying mechanisms, in order to guide the clinician in the prognostic judgment and tailoring of treatment.
